# Structure and Binding Interface of the Cytosolic Tails of αXβ2 Integrin

**DOI:** 10.1371/journal.pone.0041924

**Published:** 2012-07-26

**Authors:** Geok-Lin Chua, Xiao-Yan Tang, Alok Tanala Patra, Suet-Mien Tan, Surajit Bhattacharjya

**Affiliations:** School of Biological Sciences, Nanyang Technological University, Singapore, Singapore; Dresden University of Technology, Germany

## Abstract

**Background:**

Integrins are signal transducer proteins involved in a number of vital physiological processes including cell adhesion, proliferation and migration. Integrin molecules are hetero-dimers composed of two distinct subunits, α and β. In humans, 18 α and 8 β subunits are combined into 24 different integrin molecules. Each of the subunit comprises a large extracellular domain, a single pass transmembrane segment and a cytosolic tail (CT). The CTs of integrins are vital for bidirectional signal transduction and in maintaining the resting state of the receptors. A large number of intracellular proteins have been found to interact with the CTs of integrins linking integrins to the cytoskeleton.

**Methodology/Principal Findings:**

In this work, we have investigated structure and interactions of CTs of the leukocyte specific integrin αXβ2. We determined the atomic resolution structure of a myristoylated CT of αX in perdeuterated dodecylphosphocholine (DPC) by NMR spectroscopy. Our results reveal that the 35-residue long CT of αX adopts an α-helical conformation for residues F4-N17 at the N-terminal region. The remaining residues located at the C-terminal segment of αX delineate a long loop of irregular conformations. A segment of the loop maintains packing interactions with the helical structure by an extended non-polar surface of the αX CT. Interactions between αX and β2 CTs are demonstrated by ^15^N-^1^H HSQC NMR experiments. We find that residues constituting the polar face of the helical conformation of αX are involved in interactions with the N-terminal residues of β2 CT. A docked structure of the CT complex indicates that a network of polar and/or salt-bridge interactions may sustain the heteromeric interactions.

**Conclusions/Significance:**

The current study provides important insights into the conservation of interactions and structures among different CTs of integrins.

## Introduction

Integrins are heterodimeric cell surface receptors that mediate cell attachment and migration, and they modulate cell growth, proliferation and differentiation [1], [2]. In humans, there are 24 integrin heterodimers that are categorized into subfamilies based either on the specific-pairing of the α and β subunits or their ligands. Each integrin subunit has a large ectodomain and a transmembrane domain followed by a cytoplasmic tail (CT). Integrin ligand-binding is mediated by its ectodomain while its cytoplasmic tail allows docking of cytosolic proteins, many of which have been shown to regulate integrin ligand-binding via long-ranged allostery or to induce integrin-derived cellular signaling [2]. The β2 integrins are expressed exclusively in leukocytes and there are four members in this subfamily, namely αLβ2 (LFA-1, CD11aCD18), αMβ2 (Mac-1, CR3, CD11bCD18), αXβ2 (p150, 95, CR4, CD11cCD18) and αDβ2 (CD11dCD18) [3]. Integrin αXβ2 is expressed primarily in myeloid cells, dendritic cells and NK cells. Integrin αXβ2 has many ligands that overlap with that of integrin αMβ2, including iC3b, fibrinogen, and denatured proteins [3]. Notably, integrin αXβ2 has been shown to bind exposed negatively charged residues in decayed proteins, suggesting that it plays a role in neutrophil migration and pericellular degradation of extracellular matrix [4]. High-fat diet induced less adipose tissue inflammation in integrin αX^−/−^ knockout mice compared with wild-type mice [5]. Further, double-knockout mice (integrin αX^−/−^ and apoE^−/−^), but not apoE^−/−^ mice, showed less accumulation of macrophages in atherlosclerotic lesions [6]. These observations are in line with integrin αXβ2 as a phagocytic receptor and its role in monocyte adhesion to endothelium [7]. Integrin αXβ2 also serves as a marker to distinguish between two populations of HLA-DR+ human peripheral blood dendritic cells [8]. Although integrins do not possess enzymatic activity, they can trigger intracellular signaling by recruiting cytosolic proteins to their cytoplasmic tails aforementioned. Except for their juxtamembrane regions, the integrin α CTs are divergent in lengths and sequences [3]. There are many lines of evidence that suggest the α CTs in mediating integrin signaling specificity [9]–[12]. However, structural information of these integrin α CTs is needed to define the underlying mechanisms. Previously, we have reported the solution structures of integrin αL and αM CTs [13], [14]. Here we report for the first time the structure of integrin αX CT. Considering that the structure of the entire integrin αXβ2 ectodomain has been recently solved [15], our data will allow better understanding of integrin αXβ2 function and regulation as a whole.

## Results

### NMR Analyses of the Myristoylated CT of αX

**Figure 1:**
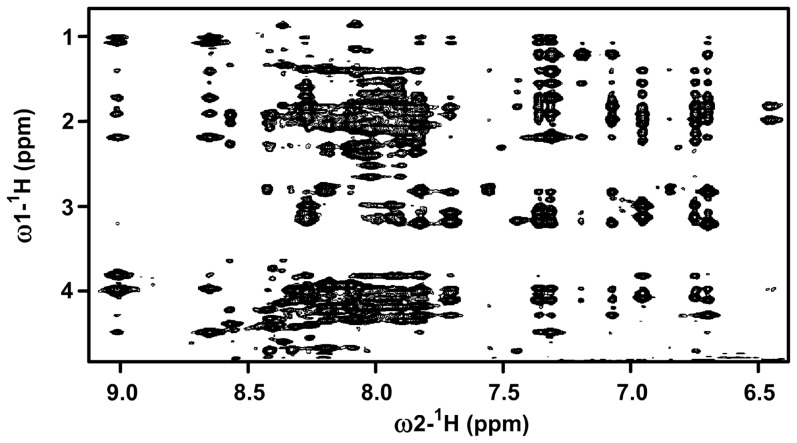
Folded conformation of myristoylated αX CT. A section of the two-dimensional ^1^H-^1^H NOESY spectrum of αX CT in DPC micelles showing NOE contacts among low-field resonances (6.5 ppm–9.0 ppm) with up-field resonances (0.8 ppm–4.5 ppm). A large number of NOE cross-peak indicates αX CT is well folded in DPC micelles. NOESY spectra were acquired in 200 mM DPC dissolved in 10 mM sodium phosphate buffer, pH 5.6, 308 K.

CTs of integrin, particularly the N-terminal region, are closely localized to the membrane through the transmembrane helical domain. We have prepared an N-terminal myristoylated CT of αX that may act as a probable mimic of transmembrane segment. Such strategy was successfully used for the structure determination of the 20-residue CT of αIIb of platelet integrin αIIb/β3 [16] and more recently for the 24-residue CT of αM of integrin αM/β2 [14]. It may be noted that fatty acylated CTs of integrins were found to be biologically active under *in vivo* studies [17]. 3-D structures of the myristoylated CTs were obtained in zwitterionic DPC micelles by NMR spectroscopy [14], [16]. Despite the longer length of the αX CT in comparison to that of CTs of αIIb and αM well resolved NMR spectra were observed in DPC micelles. Figure 1 shows a section of 2-D ^1^H-^1^H NOESY spectrum of the myristoylated αX CT in DPC micelles correlating downfield shifted amide and aromatic proton resonances at 6.6–9.0 ppm with the upfield shifted aliphatic proton resonances 4.5–0.9 ppm. Observation of a large number of NOE correlations among the amide proton resonances (7.8–9.0 ppm) and aliphatic resonances indicates that CT adopts a well defined conformation in DPC micelles. The aromatic ring protons resonating at 6.8–7.4 ppm delineates NOE correlations with the upfield shifted resonances at 0.9–2.5 ppm of aliphatic sidechain protons, indicating a close proximity of these aromatic and alkyl sidechains (Figure 1). The sequence-specific resonance assignments of the CT of αX in DPC were achieved by the combined use of 2-D TCOSY and NOEST spectra.

**Figure 2:**
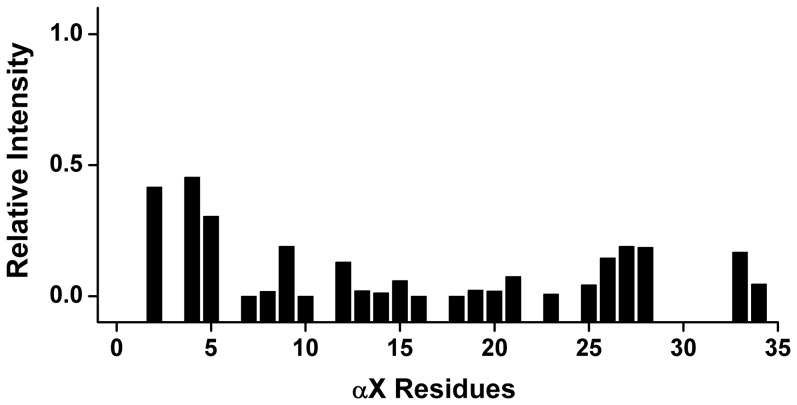
Localization of αX CT in DPC micelles by PRE. A bar diagram showing ratio of intensity of NH/CαH correlation obtained from ^1^H-^1^H TOCSY spectra of αX CT in the absence and in the presence of 1 mM MnCl_2_. As can be seen, most of the residues of αX CT had experienced a marked quenching (low value in intensity ratio) in the presence of paramagnetic Mn^2+^, suggesting solvent exposure of the CT.

We carried out paramagnetic relaxation enhancement (PRE) experiments with MnCl_2_ to determine the localization of residues of αX CT in DPC micelles. In particular, 2D NOESY spectra of the CT in DPC micelles were acquired in the presence of 1 mM MnCl_2_. It may be noted that paramagnetic Mn^2+^ would enhance the relaxation of resonances of those that are exposed to aqueous environment. Figure 2 shows residual intensity of NH/CαH NOESY cross-peaks as a function of amino acid residues of αX CT. As one would expect, there was a marked diminution of the intensity of NH/CαH correlations for most of the residues of αX CT; indicating their close proximity to the paramagnetic Mn^2+^ ions (Figure 2). However, a relatively higher residual intensity of NH/CαH cross-peaks can be seen for some of the N-terminal residues namely for G3, F4 and F5 (Figure 2). This data may suggest a probable partial inclusion of these hydrophobic residues within the lipid region of DPC micelles (Figure 2). Taken together, PRE studies established that most of the residues of αX CT are predominantly located in the aqueous milieu.

### Chemical Shifts Deviations and Nuclear Overhauser Effects

**Figure 3:**
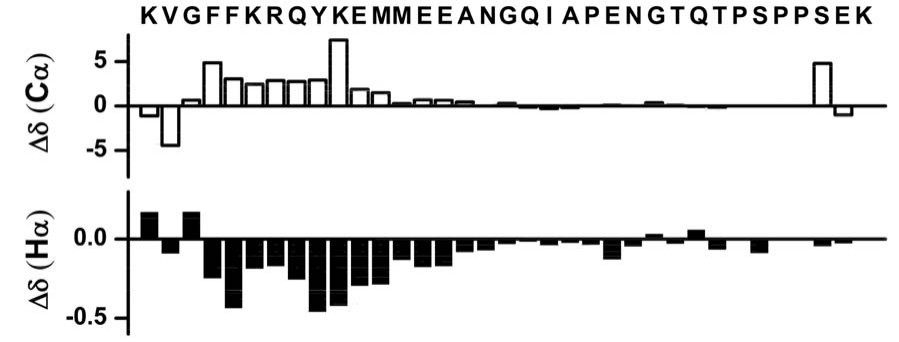
Secondary structure of the αX CT from chemical shift deviations. Bar diagrams representing deviation of ^13^Cα (top panel and CαH (bottom panel) chemical shifts from random coil values for amino acid residues of αX CT in DPC micelles.

**Figure 4:**
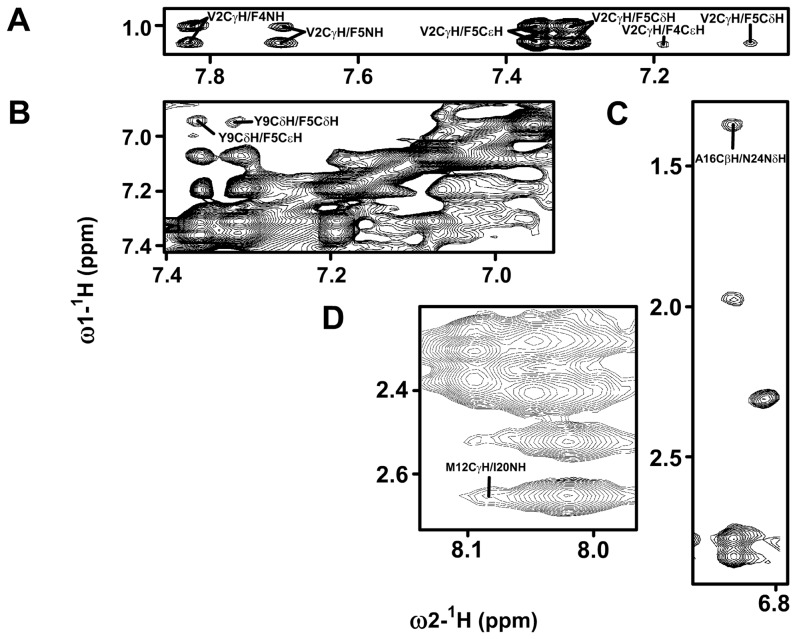
Packing interactions in the αX CT in DPC micelles. Selected regions of two-dimensional ^1^H-^1^H NOESY spectra of the αX CT showing NOE contacts among; (panel A) aromatic ring proton resonances with the aliphatic sidechain resonances, (panel B) aromatic ring proton resonances of F5 and Y9, (panels C and D) sidechains of M12/I20 and A16/N24.

Secondary structures of amino acids in peptides and proteins can be identified from the deviation of chemical shifts of αH and ^13^Cα nuclei from the random coil values [18]. Figure 3 shows secondary chemical shifts of ^13^Cα (top panel) and αH (bottom panel) of each amino acid of αX CT in DPC micelles. Residues F4-A16 appeared to be experiencing a positive deviation for ^13^Cα and a negative deviation for αH chemical shifts, indicating helical conformations for this segment. By contrast, secondary chemical shift for the residues N17-K35 were not conspicuous, suggesting a loop or random conformations. Helical conformations for F4-A16 were further deduced from the medium range NOE contacts involving diagnostic CαH/HN (i to i+2, i+3 and i+4) and NH/NH (i to i+1 and i+2) resonances. The helical conformation for residues F4-A16 of αX CT was also defined by a number of medium range NOEs of sidechain/sidechain and backbone/sidechain (Figure 4). NOEs could be observed among the aromatic ring protons of F4 and Y9 (Figure 4A) and among the aliphatic sidechain protons of M12 with aromatic sidechain of Y9 (Figure 4B). A few long-range NOEs were detected between residues from the loop with residues of helix (Figure 4 panels D and E). In particular, M12 CγHs and A16 CβH_3_ showed NOE contacts with amide proton and sidechain protons of I20 and N24. Taken together, secondary chemical shifts and NOE contacts establish that the myristoylated αX CT in DPC micelles assumes a well folded helical conformation for residues F4-A16 at the membrane proximal region followed by less defined secondary conformations for the C-terminus part. However, the C-terminus loop appears to retain a definite orientation through its packing interactions with the helical segment.

### Three-dimensional Structure of the CT of αX in DPC Micelles

**Figure 5:**
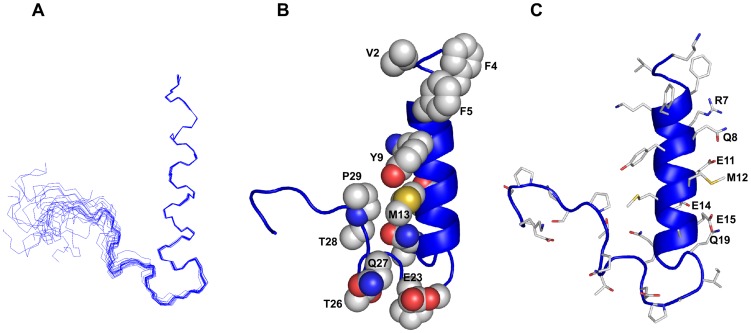
Three-dimensional structure of the αX CT in DPC micelles. (Panel A) Superposition of backbone atoms (N, Cα, C′) of twenty lowest energy conformers of the αX CT in DPC micelles. (panel B) A space-filling representation of the αX CT structure showing sidechain-sidechain packing interactions between residues from the helix and the loop. (panel C) A representative structure of the αX CT depicting sidechain orientation. Figures were generated by use of PYMOL.

NMR structures of αX CT were determined based on 263 NOE-driven distance constraints and 57 backbone dihedral constrains (Φ, Ψ) using CYANA (Table 1). Figure 5A shows a superposition of all backbone atoms (Cα, N, and C′) of an ensemble of twenty lowest energy structures of αX CT. The RMSD values from the mean structure and the stereo-chemical goodness of the structural ensembles are listed in Table 1. The αX CT is defined by a membrane proximal N-terminal α-helical conformation encompassing residues F4-N17 (Figure 5, panels B and C). The propagation of helical conformation appeared to be terminated at residue G18 that assumes a left-handed helical conformation with a positive value in backbone dihedral angles. The helical structure demonstrates an amphipathic organization of the sidechain of amino acids (Figure 5, panels B and C). In particular, one face of the helix is highly polar/ionic with residues R7, Q8, K10, E11, E14, E15 (Figure 5C). The sidechain of residue Q19, though non-helical, also points towards the hydrophilic side of the helix (Figure 5C). The hydrophobic face of the helix of αX CT is defined by packing among aromatic and aliphatic sidechain of residues V2, F5, Y9 and M13 (Figure 5B). A part of the C-terminus residues i.e. Q19-T26 displays well defined turn-like conformations as indicated by the close superposition of the structural ensemble for these residues (Figure 5A). There are long-range packing interactions among residues from the turn region with the hydrophobic face of the helix (Figure 5B). In particular, an extended non-polar surface of αX CT can be realized by the mutual packing of residues Y9, M13 of the helix with residues Q27, T28 and P29 in the turn (Figure 5B). By contrast, other C-terminal residues S30-K35 are found to be rather disordered in the NMR structure of αX CT (Figure 5A).

**Table 1:** Summary of structural statistics of the twenty lowest energy structures of myristoylated αX CT in DPC micelles.

**Distance restraints**	Intra-residue (|i−j| = 0)	74
	Sequential (|i−j| = 1)	90
	Medium range (2≤|i−j|≤4)	96
	Long-range (|i−j|≥5)	3
**Dihedral angle constraints (Φ, Ψ)**		57
**Constraints violations**	Average NOE violation (Å)	0.25
	Maximun NOE violation (Å)	0.31
a **Deviation from mean structure**	Backbone atoms (N, Cα, C′) (Å)	1.14 (0.11)
	Heavy atoms (Å)	1.89 (1.14)
**Ramachandran plot analysis**	% residues in the most favorable region	82
	% residues additionally allowed region	18
	% residues in the generously allowed region	0
	% residues in the disallowed region	0

a The RMSD values for the N-terminal helical region (residue 2–17) are in parentheses.

### Mapping Binding Residues of αX and β2 CTs by ^15^N-^1^H HSQC

**Figure 6:**
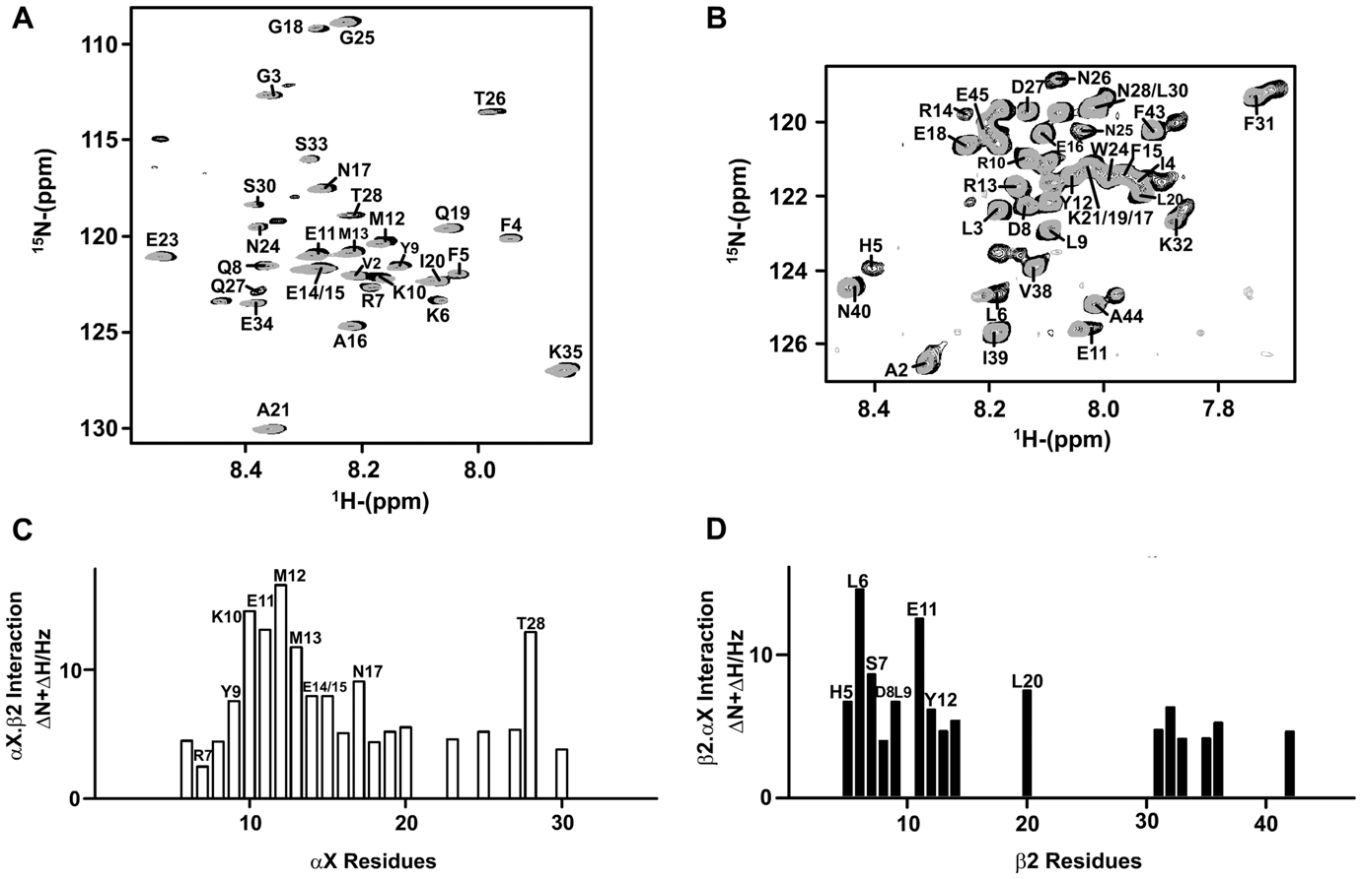
Interactions between αX CT and β2 CT by ^15^N-^1^H HSQC NMR. (panel A) Selected section of ^15^N-^1^H HSQC spectrum of ^15^N labeled αX CT in free (in black contour) and in the presence of unlabelled β2 CT (in grey contour) at a ratio of 1∶2 (αX: β2). (panel B) Selected section of ^15^N-^1^H HSQC spectrum of ^15^N labeled β2 CT in free (in black contour) and in the presence of unlabelled αX CT (in grey contour) at a ratio of 1∶2 (β2: αX). (panel C) A bar diagram showing combined chemical shift changes of ^15^N and ^1^HN resonances (in Hz) of αX CT as a function of amino acid residues. (panel D) A bar diagram showing combined chemical shift changes of ^15^N and ^1^HN resonances (in Hz) of β2 CT as a function of amino acid residues.

Interactions between the CTs of αX and β2 were probed by obtaining ^15^N-^1^H HSQC spectra of the ^15^N-labeled CT samples in the presence unlabelled binding partner in aqueous buffer solutions. The ^15^N-^1^H HSQC spectrum of αX CT was assigned by the use of stranded triple resonance NMR experiments (see materials and methods). The HSQC spectrum of β2 CT was previously assigned [13]. Figure 6A shows overlay of the ^15^N-^1^H HSQC spectra of the αX CT in free (black contour) and after addition of CT of β2 (gray contour) at a 1∶2 molar ratio. Chemical shift changes of amide proton and ^15^N resonances could be detected for several residues in the HSQC spectra of the αX CT in the presence of β2 CT, indicating interactions between the CTs. In reverse titrations, chemical shift changes were also observed in the ^15^N-^1^H HSQC spectra of the β2 CT upon addition of the cognate CT of αX (Figure 6B). The changes in chemical shifts are summarized in Figures 6C and 6D for the αX CT and for the β2 CT, respectively. As can be seen, residues Y9, K10, E11, M12, M13, E14/E15, N17 and T28 of the αX CT demonstrated discernable changes in chemical shifts in comparison to other residues, indicating their probable involvement in binding with the β2 CT (Figure 6C). Notably, most of the residues, K10, E11, M12, E14, E15 and N17, of αX CT displaying binding induced chemical shift perturbation occupy the hydrophilic face of the amphipathic helix. By contrast, residues from the loop region of αX CT delineated limited chemical changes, indicating lack of binding interactions with the β2 CT (Figure 6C). However, a lone residue T28 from the loop appeared to exhibit chemical shift changes akin to the helical residues (Figure 6C). Interestingly, T28 among the loop residues that has packing interactions with the helical structure (Figure 5B). Perhaps, binding of β2 CT with the helical region of αX CT might have influenced the packing interactions between the loop and the helix. For the β2 CT, pronounced chemical shift and/or intensity changes were observed for the N-terminal residues H5, L6, S7, D8, L9, E11, Y12 and R14. The HSQC cross-peaks of H5 and R14 cannot be detected in the presence of αX CT, presumably as a result of conformational exchange between the free and bound states (Figure 6B).

### Molecular Models of the Complex αX/β2 CTs

**Figure 7:**
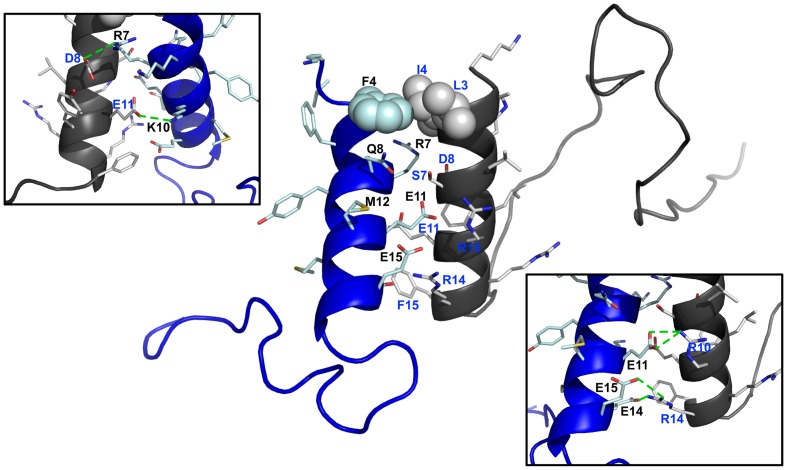
Docked structure of the αX/β2 CTs. Plausible interfacial residues of the complex of CTs of αX/β2 integrin. The polar face of the amphipathic helical structure of αX CT (in blue ribbon) appears to be engaged in multiple ionic and/or hydrogen bond interactions with the membrane proximal helix of β2 (in black ribbon). The probable mutual sidechain-sidechain packing among the non-polar and aromatic residues of αX CT with the β2 CT at their N-termini is represented by space filling.

Based on changes of chemical shifts, energy-refined docked structures were generated, using RosettaDock protocol [19], of the complex of αX and β2 CTs. The N-terminus of the β2 CT has been determined to assume helical structure in our previous study [13]. A complex formation between the two CTs could be potentially sustained by a number of salt-bridge and polar interactions along with hydrophobic packing through a parallel orientation (Figure 7). In particular, the docked structure of the hetero-tail complex revealed close proximity between the sidechain of residues R7, K10, E11 of the αX CT with the sidechain of residues of D8, E11 and R10 of β2 CT, respectively (Figure 7). Further, the sidechain of residues R10 and R14 of β2 CT may form multiple H-bonds and/or salt bridges with the anionic sidechain of E14 and E15 of αX CT (Figure 7). In addition, polar residues S7 of β2 CT and Q8 of αX CT are in close proximity in the docked structure. A patch of non-polar packing interactions is probable between the two helices involving aromatic ring of F4 of αX and alkyl chain of L3 and I4 of β2 CT (Figure 7). Collectively, the tail-tail hetero-complex between αX and β2 subunits of αX/β2 integrin may be stabilized by interactions of membrane proximal helices, whereas the residues at the long loop region of αX or β2, situated far from the interface, are amenable for binding with other cytosolic proteins.

## Discussion

**Figure 8:**
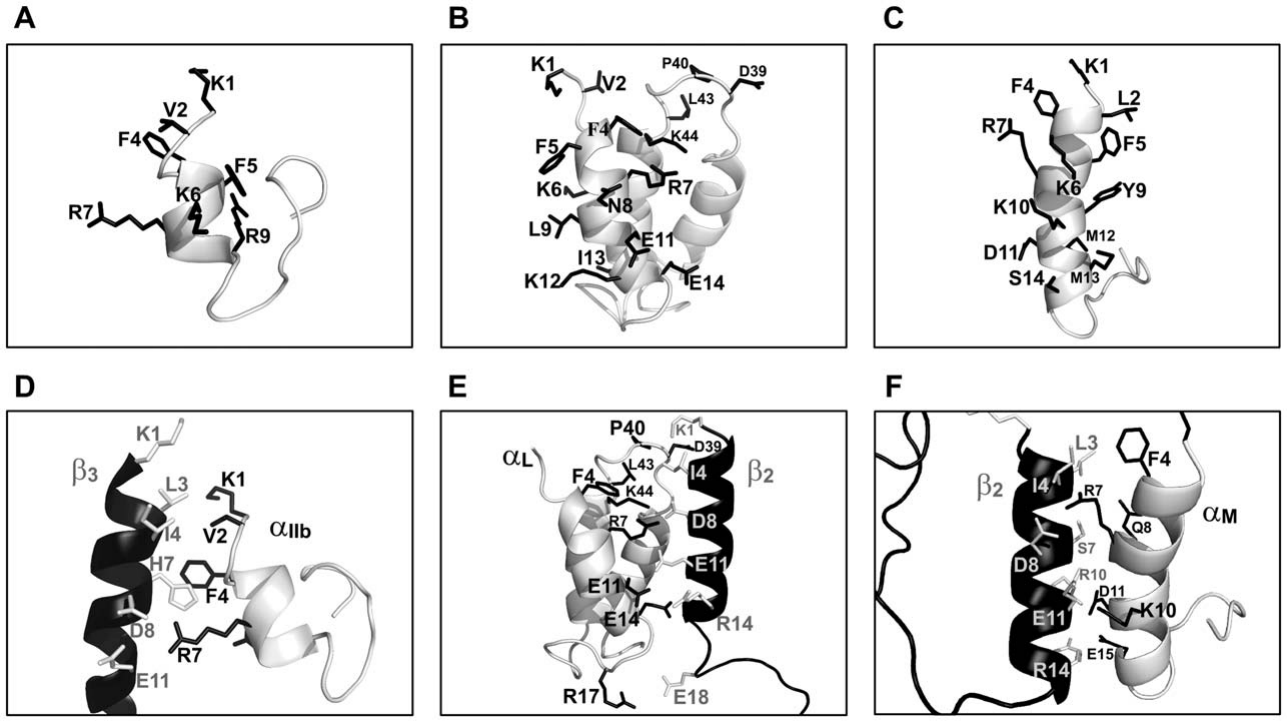
Structures and interactions of the α CTs of integrins. (top panel) Ribbon representation of the 3-D structures of different a CTs determined in previous studies (panel A) αIIb CT of αIIb/β3 integirn, (panel B) αL CT of αLβ2 integrin, (panel C) αM CT of αMβ2 integrin. (bottom panel) Comparison of the complexes of α and β CTs of (panel A) αIIb/β3 integirn, (panel B) αLβ2 integrin and (panel C) αMβ2 integrin.

The CTs of integrins are involved in bidirectional signaling by interacting with cytoplasmic proteins [1]–[3]. Most of the integrin β-subunits have CTs that are well conserved with sequence motifs NPXX(Y/F) binding to talin, kindlin and DOK proteins. By contrast, integrin α-subunits have CTs that are less conserved, except for the membrane proximal region (Figure S1). Notably, different α CTs exhibit specific interactions with cytoplasmic proteins namely α5 with nischarin [20], α4 with paxillin [21], αIIb with calcein integrin binding protein [22], and αL with CD45 cytoplasmic domain [23]. Conceivably, interactions between α CTs and the cytosolic binding partners may dictate specific function of integrins. Thus, structural elucidation of various α CTs could be useful not only to gain insights into integrin regulation but also for the development of specific anti-integrin drugs [24], [25]. To-date, structures are known for three α CTs namely αIIb/β3, αLβ2 and αMβ2 integrins [13], [14], [16] (Figure 8, top panel). The current study defined the 3-D structure of the myristoylated αX CT in DPC micelles. The αX CT demonstrates a folded structure characterized by the N-terminal amphipathic helix and a distal loop akin to the CTs of αM and αIIb integrins. However, there are striking differences between the structures of the αX CT and the αIIb CT in terms of the length of the N-terminal helix and molecular contacts of the distal loop with the helix. The NMR structure of the myristoylated integrin αIIb CT which is 20-residue long, is characterized by a short helix (V^990^-R^997^) and an acidic loop (E^1001^EDDEEGE^1008^) (Figure 8A). The acidic loop binds to metal ions and folds back onto the helix by salt-bridge interactions [16]. By contrast, the N-terminal helix of αX CT is considerably longer involving residues F4-N14, and it is also amphipathic (Figure 5). The hydrophilic or polar face of the amphipathic helix of αX CT is well characterized by an array of possible salt-bridge, (residues R7-E11, K10-E14) and hydrogen bond (residues E14-Q19), interactions. The membrane proximal helix of the αIIb CT is rather hydrophobic with fewer polar interactions. In addition, the acidic loop residues of the αIIb CT acquires a tighter packing with the helix by ionic interactions. On the other hand, the long distal loop, residues I21-K37, of the αX CT experiences a higher degree of conformational variations. Only the first few residues of loop of αX CT is involved in packing interactions with the hydrophobic face of the N-terminal helix. The other residues of the loop of αX CT remains extended. Recently, we have determined NMR structure of the myristoylated αM CT of integrin αMβ2 in DPC micelles [14]. The N-terminal region of the 24-residue long αM CT adopted an amphipathic helix, residue F4-E15, followed by a short, residues G15-Q23, fold back loop [14] (Figure 8B). By contrast, the 3-D structure of 57-residue long αL CT was defined by mutual packing of three helices with inter-connecting loops (Figure 8C). The folded conformation of αL CT, sustained by salt bridges and/or hydrogen bonds, display a large negatively charged surface that is involved in binding to metal ions [13]. Recently, NMR structures have been solved for the TM domain of αIIb either with the full-length or C-terminal truncated CT under different solution conditions [26], [27]. A helical conformation for the membrane proximal region has been deduced for the full-length CT in the context of the TM domain in a membrane mimetic organic solvent-water mixture [26]. By contrast, NMR structure, in lipid bicelles, of the TM with truncated CT of αIIb revealed a bent or reverse turn conformation for the membrane proximal segment packing with the TM helix [27]. The structural disparity of the membrane proximal region of the αIIb CT noted in these studies may either simply result from the differences in constructs used or an indicative of favored mode of the structural intermediates. Further, NMR derived structure of the full-length TM and CT of α1 integrin shows helical structure for the membrane proximal region of CT [Lai C et. al. unpublished results, pdb accession number 2L8S]. Aforementioned studies including our current results, therefore, suggest that the membrane proximal region of the full-length α CTs of various integrins assumes a conserved helical structure. However, the C-terminal regions of α CTs appear to show a marked variability in their conformations. It is tempting to speculate that such conformational disparity may allow binding of α CT specific cytosolic proteins resulting in various signaling outcomes.

Integrins undergo complex structural changes from a closed bent conformation to an extended open conformation when activated [1]–[3]. Mutagenesis and FRET studies in full length integrins have shown interactions between the CTs of α and β subunits [28]–[30]. However, analyses of interactions between isolate CTs of αIIb/β3 integrin were not unambiguous. NMR structure has been determined for the αIIb and β3 CTs complex [31], but, initial studies were not able to detect binding between the CTs, probably due to weak interactions [32], [33]. A recent study has demonstrated interactions between αIIb and β3 CTs in DPC micelles whereby a disulfide bond was introduced utilizing a short segment of the TM domains [34]. These results showed conformational stabilization of the β3 CT in lipid environments influencing its interactions with cognate αIIb CT [34]. Along this line, membrane tethering of the β-CT of αM β2 integrin has been found to increase the affinity of interactions between the CTs [14]. We have demonstrated that CT of αL and αM interacts with the cognate CT of β2 by NMR spectroscopy [13], [14]. However, the magnitude of chemical shift perturbation was found to be limited indicting weak binding affinity between the free CTs. In this work, we observed interactions between the αX CT and β2 CTs. ^15^N-^1^H HSQC results revealed that binding residues are located at the conserved membrane proximal helical segment of αX and β2 CTs (Figure 6). The docked structure between the CTs of αX and β2 reveals an interface that is predominantly sustained by a large number of polar and ionic interactions (Figure 7). Previous studies have deduced residues at the interface contact between CTs of αIIb/β3, αL/β2 and αM/β2 integrins (Figure 8, D, E, F). All of these interfaces including αXβ2 have a conserved inter-subunit salt-bridge between R7 of α CT and D8 of β CT at the membrane proximal region (Figure 7 and Figure 8). However, the complex between the CTs of αIIbβ3 integrin delineates much shorter interfacial contacts in comparison to that of CTs, including αXβ2, of leukocyte integrins (Figure 8D). The docked structure of αX and β2 CTs suggests a plausible extended interfacial region distal to the membrane proximal helices. It is likely that these interactions between CTs of leukocyte integrins may stabilize the resting state of these integrins. In order to validate the current model of the complex between CTs of leukocyte integrins, we plan to carry out NMR structural studies of the integrin segments that contain the CT and TM.

## Materials and Methods

### Synthesis and purification of myristoylated αX tail

The myristoylated form of the αX CT was purchased in the crude form from GL Biochem (Shanghai, China). The crude peptide was purified by a linear acetonitrile/water gradient, at a flow rate of 2 ml/min, passing through a C18 column (300 Å pore size, 5 µM particle size) connected to a Waters reverse phase High Performance Liquid chromatography (HPLC) system (Massachusetts,USA). The major peak fraction was collected and lyophilized. The peptide was later reconstituted in the required buffers.

### Expression and purification of αX and β2 cytosolic tails

The CTs of β2 (residues K724-S769) and αX (residue K1129-K1163) were sub-cloned into the pET-31b(+) vector to generate fusion proteins containing N-terminal ketosteroid isomerase (KSI). Fusion proteins containing KSI were expressed in inclusion body in *E. coli* resisting proteolysis of the target proteins [13], [14]. The sub-cloning and expression of the β2 CT was reported in our previous works [13], [14]. In this study, αX CT was sub-cloned into a pET-31b(+) vector (Novagen EMD, San Diego) from αX-pcDNA3.0 expression plasmid. A formic acid-cleavage GGGGSDP sequence was introduced between the KSI and the αX CT sequences that enables formic acid digestion at the DP site of the αX CT from the KSI fusion protein [14]. The pET-31b vector was also modified containing an N-terminal six His residues (his-tag) and a stop codon immediately after the residue K1163 of αX CT to avoid the expression of a C-terminal his-tag from the original vector. Thereby, the αX CT can be purified without any additional tag sequence from the vector. Purification and isotope labeling, ^15^N and ^15^N/^13^C, of the CTs were carried out as described previously [13], [14]. Briefly, *E. coli* BL21DE3 cells (New England Biolabs Inc.), containing expressing plasmids, were cultured either in rich LB medium or in minimal media containing ^15^N, ammonium chloride or ^15^N ammonium chloride and ^13^C glucose at 37°C in shaking incubator at 150 rpm. Expression of fusion proteins were induced by isopropyl β-D-1-thiogalactopyranoside (IPTG), 1 mM concentration, at a cell density of 0.7–0.9. The induced cell cultures were kept at 25°C for 18 hours for protein production in an incubator with a shaking speed of 150 rpm. The cells were then collected, via centrifugation at 5000 rpm for 20 mins, and resuspended in 20 mM Tris-Cl, 0.5 M NaCl buffer, pH 8.0. The resuspended cells were then lysed via sonication on ice to release the cellular contents that contain the recombinant fusion proteins. As the KSI fusion proteins were targeted to inclusion bodies, the cell pellets were collected via centrifugation at 14 000 rpm for 30 mins and re-solubilized using a buffer containing 20 mM Tris-Cl, 0.5 M NaCl and 8 M urea, pH 8.0 to release recombinant proteins. The supernatant containing the solubilized KSI fusion protein was affinity purified using Nickel-NitriloTriAcetic (NTA) acid (QIAGEN) beads by making use of the his-tag attached to fusion proteins. The KSI fusion proteins were then eluted with 20 mM Tris-Cl, 0.5 M NaCl and 8 M urea buffer pH 8.0 with 0.5 M imidazole. The eluates were dialyzed against water at 4°C for 2 days to remove urea, causing the formation of KSI-protein precipitates, which were collected by centrifuging at 5000 rpm for 30 mins. KSI-αX fusion protein was cleaved with 90% formic acid at a ratio of 1 mg KSI-αX to 1 ml of 90% formic acid, purged with N_2_ gas to cleave the Asp-Pro connector between KSI and CT of αX for 22 hrs in the dark. The CT of β2 was obtained by treating fusion protein, dissolved in 70% formic acid, with cyanogen bromide. The reaction was also purged in N_2_ gas and left in the dark for 22 hrs. For both reactions, formic acid and/or cyanogen bromide were neutralized with sodium hydroxide using a rotary evaporator, leaving a film of precipitate. The precipitate was dissolved in 10 mM sodium phosphate buffer pH 6.5 and further purified using HPLC. The identities of the cleaved peptides were verified with mass spectrometry.

### NMR Experiments

NMR experiments were carried out on a Bruker DRX 600-MHz spectrometer equipped with an actively shielded cryo-probe. 10% deuterium oxide and 2 mM 2,2-dimethyl-2-silapentane-5-sulfonate (DSS) were added to all NMR samples. All NMR spectra are referenced to the ^1^H of DSS. 2-dimensional ^1^H-^1^H Total Correlation Spectroscopy (TOCSY) and Nuclear Overhauser Spectroscopy (NOESY) experiments were carried out at 308 K for 0.7 mM of myristoylated CT of αX dissolved in 10 mM sodium phosphate buffer pH 5.6, containing 200 mM deuterated dodecylphosphocholine (DPC) (CIL, Massachusetts, USA). TOCSY mixing time was fixed at 50 ms and NOESY mixing time was kept at 200 ms. In addition, natural abundance ^13^C-^1^H Heteronuclear Single Quantum Coherence (HSQC) experiment was carried to obtain ^13^Cα chemical shifts for the myristoylated peptide. NMR data was processed using TOPSPIN 2.1 and analyzed with SPARKY [35]. The NOESY spectra were assigned with the aid of the TOCSY spectrum using the sequence specific resonance assignment strategy. Paramagnetic relaxation enhancement experiments were carried out by collecting 2-dimensional TOCSY spectra of myristoylated αX CT dissolved in 10 mM sodium phosphate buffer containing 200 mM deuterated DPC, in the absence and presence of 1 mM MnCl_2_. The intensity of the intra-residue CαH/NH cross-peaks after the addition of MnCl_2_ was normalized to the peak intensity of the unperturbed sample. Triple resonance 3-D HNCACB and 3-D CBCA(CO)NH experiments were performed on 0.5 mM of ^15^N/^13^C αX CT dissolved in 10 mM sodium phosphate buffer pH 6.5 at 298K, to obtain the assignment of backbone resonances. 2-dimensional ^15^N-^1^H HSQC was used to determine the interactions between the CTs of αX and β2. ^15^N-labelled CT of αX or ^15^N-labelled CT of β2 samples were dissolved in 10 mM sodium phosphate buffer, pH 6.5 and titrated with unlabeled cognate CT upto two times the concentration of the labeled sample. The changes in ^15^N and ^1^HN chemical shift were calculated using the following equation: Δ^1^H+Δ^15^N, where Δ^1^H and Δ^15^N refer to the absolute value of the change in chemical shift after addition of the binding partner.

### Structure Determination and Docking

An ensemble of conformations of myristoylated αX CT in DPC micelles was obtained by CYANA 2.1 program [36]. The NOESY cross-peaks were qualitatively translated to upper bound distance limits of 2.5 Å, 3.5 Å and 5.0 Å based on the observed signal intensity, with the stronger signal assigned a shorter distance restraint between the two protons. These distances together with the predicted backbone dihedral angles were used to carry out several rounds of structure calculation. Of the 100 structures, twenty lowest energy structures were selected for evaluation and analyses. PROCHECK-NMR [37] was employed to evaluate the stereochemical quality of the structure ensembles and figures were prepared using PYMOL, MOLMOL, and Discovery Studio Visualizer 2.0. The NMR derived structures of CTs of αX and β2 were placed in close contact to each other based on the residues perturbed in the chemical shift perturbation experiment to form the approximated starting structure for input into the RosettaDock Server [19].

## Supporting Information

Figure S1 **Comparison of primary structures of representative α and β cytosolic tails of integrins.** Alignment of amino acid sequences of α CTs (top panel) and β CTs (lower panel) of integrins αX, αM, αL, αD, αIIb and β4 and β2, β3 and β1 subunits.
(TIF)
