# Supplementary figures and images for: Structure and Binding Interface of the Cytosolic Tails of αXβ2 Integrin
Source: PLoS One. 2012 Jul 26;7(7):e41924. doi: 10.1371/journal.pone.0041924 (PMC3406025; doi:10.1371/journal.pone.0041924)

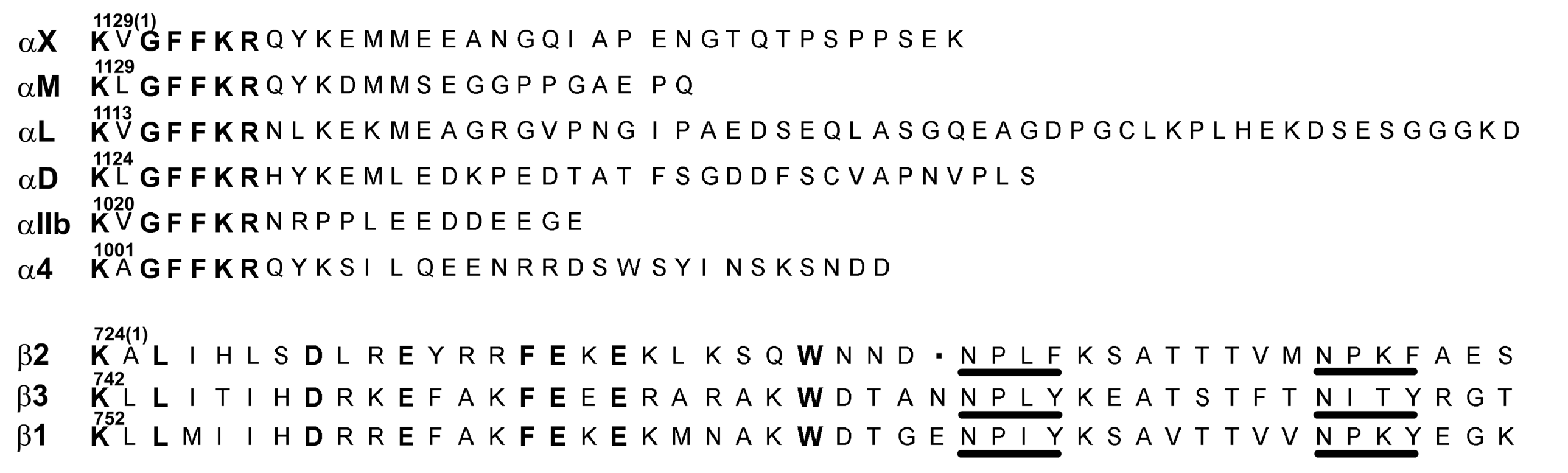

Supplement: Figure S1 — Comparison of primary structures of representative α and β cytosolic tails of integrins. Alignment of amino acid sequences of α CTs (top panel) and β CTs (lower panel) of integrins αX, αM, αL, αD, αIIb and β4 and β2, β3 and β1 subunits. (TIF) [file pone.0041924.s001.tif]
